# Supplementary figures and images for: The mitochondrial iron transporter ABCB7 is required for B cell development, proliferation, and class switch recombination in mice
Source: eLife. 2021 Nov 11;10:e69621. doi: 10.7554/eLife.69621 (PMC8585479; doi:10.7554/eLife.69621)

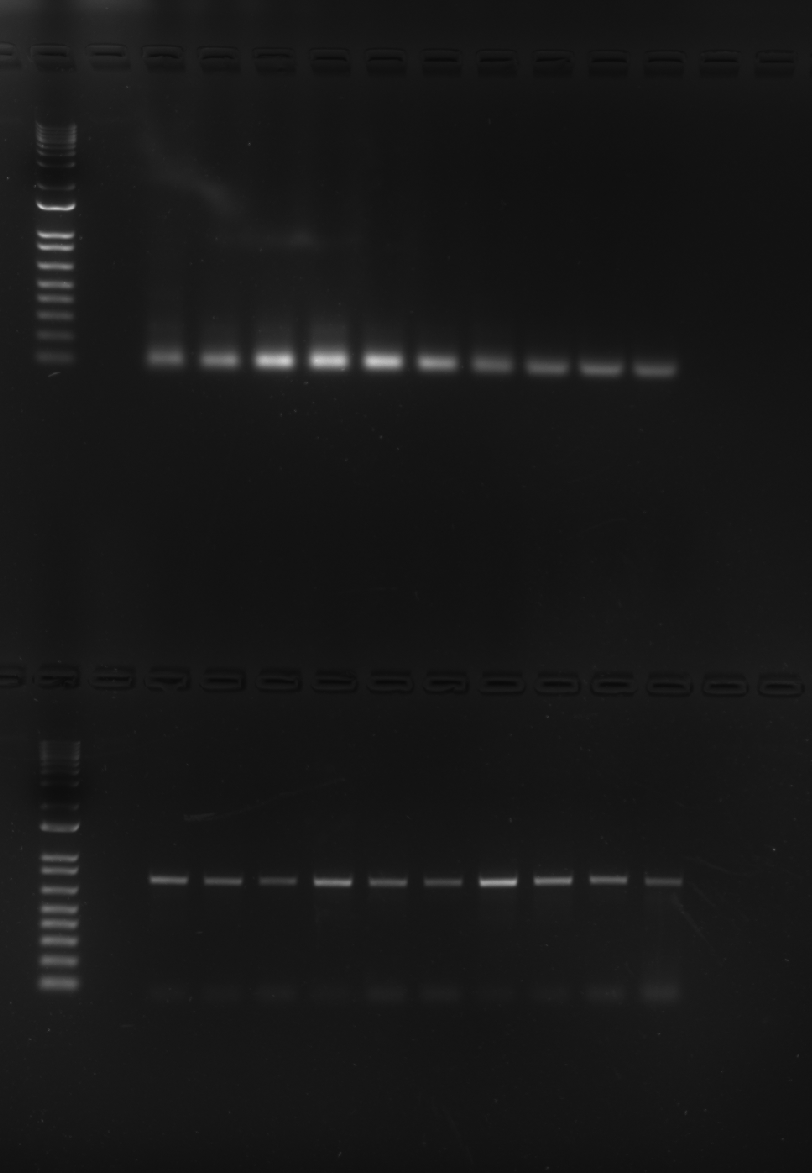

Supplement: Figure 5—source data 1. — This zip archive contains all raw gel images taken for semiquantitative PCR data shown in Figure 5B. Gels were photographed using an Omega Lum G gel imager, which saved the raw image files provided here. Individual files were named based on the VH gene family that was analyzed, and images were saved as full-resolution, 16-bit grayscale TIFF files. In addition to the unedited gel images, a labeled image is provided (named as ‘labeled’) for each gel. [file elife-69621-fig5-data1.zip › Source Data/Actin (bottom gel).tif]

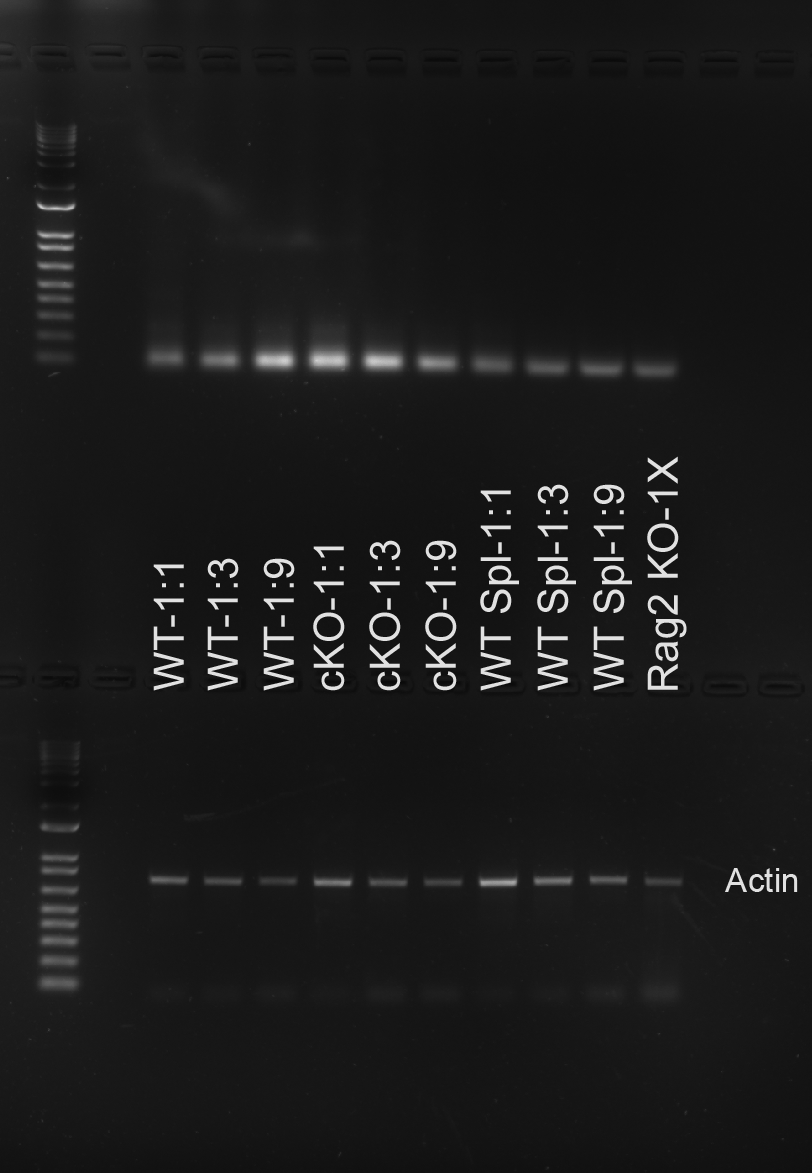

Supplement: Figure 5—source data 1. — This zip archive contains all raw gel images taken for semiquantitative PCR data shown in Figure 5B. Gels were photographed using an Omega Lum G gel imager, which saved the raw image files provided here. Individual files were named based on the VH gene family that was analyzed, and images were saved as full-resolution, 16-bit grayscale TIFF files. In addition to the unedited gel images, a labeled image is provided (named as ‘labeled’) for each gel. [file elife-69621-fig5-data1.zip › Source Data/Actin (bottom gel)_labeled.tif]

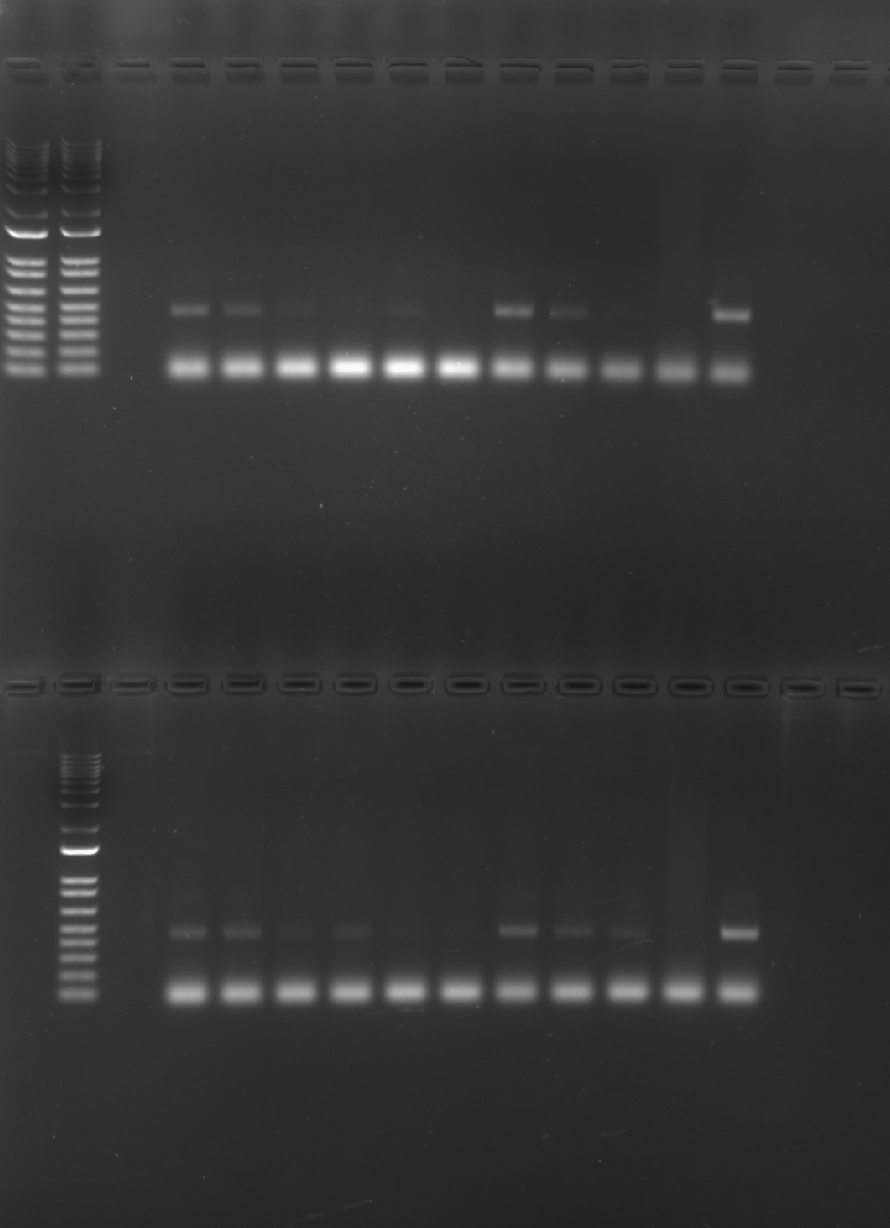

Supplement: Figure 5—source data 1. — This zip archive contains all raw gel images taken for semiquantitative PCR data shown in Figure 5B. Gels were photographed using an Omega Lum G gel imager, which saved the raw image files provided here. Individual files were named based on the VH gene family that was analyzed, and images were saved as full-resolution, 16-bit grayscale TIFF files. In addition to the unedited gel images, a labeled image is provided (named as ‘labeled’) for each gel. [file elife-69621-fig5-data1.zip › Source Data/VH3609 (bottom gel).tif]

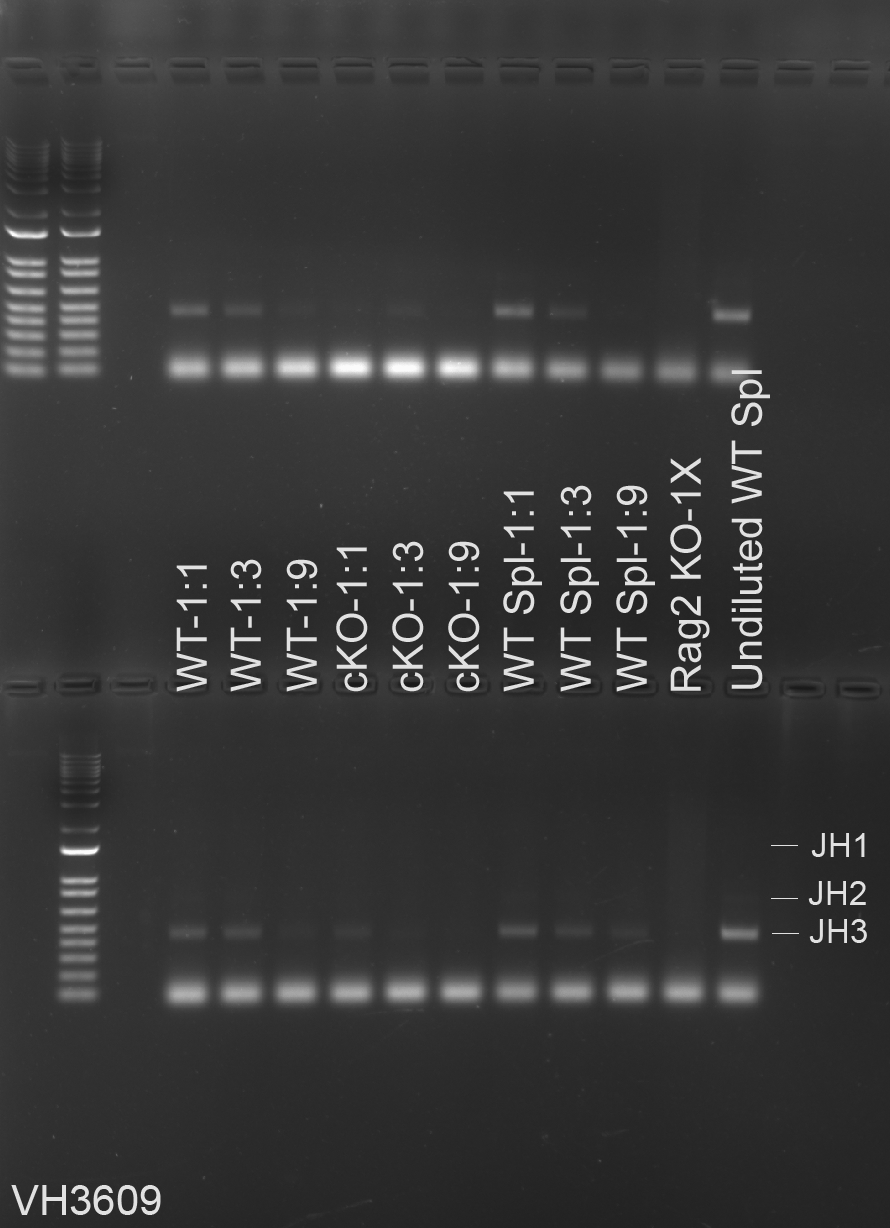

Supplement: Figure 5—source data 1. — This zip archive contains all raw gel images taken for semiquantitative PCR data shown in Figure 5B. Gels were photographed using an Omega Lum G gel imager, which saved the raw image files provided here. Individual files were named based on the VH gene family that was analyzed, and images were saved as full-resolution, 16-bit grayscale TIFF files. In addition to the unedited gel images, a labeled image is provided (named as ‘labeled’) for each gel. [file elife-69621-fig5-data1.zip › Source Data/VH3609 (bottom gel)_labeled.tif]

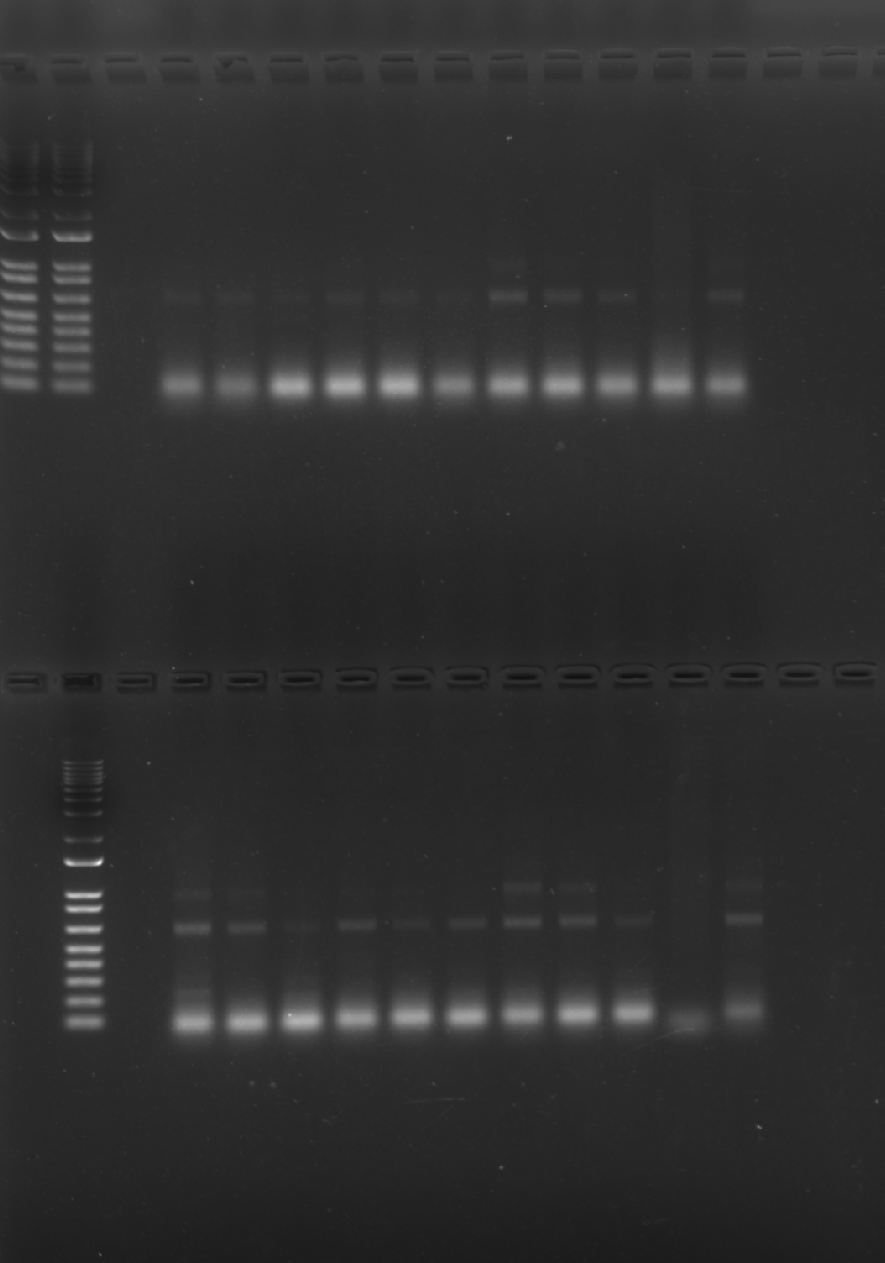

Supplement: Figure 5—source data 1. — This zip archive contains all raw gel images taken for semiquantitative PCR data shown in Figure 5B. Gels were photographed using an Omega Lum G gel imager, which saved the raw image files provided here. Individual files were named based on the VH gene family that was analyzed, and images were saved as full-resolution, 16-bit grayscale TIFF files. In addition to the unedited gel images, a labeled image is provided (named as ‘labeled’) for each gel. [file elife-69621-fig5-data1.zip › Source Data/VH7183 (bottom gel).tif]

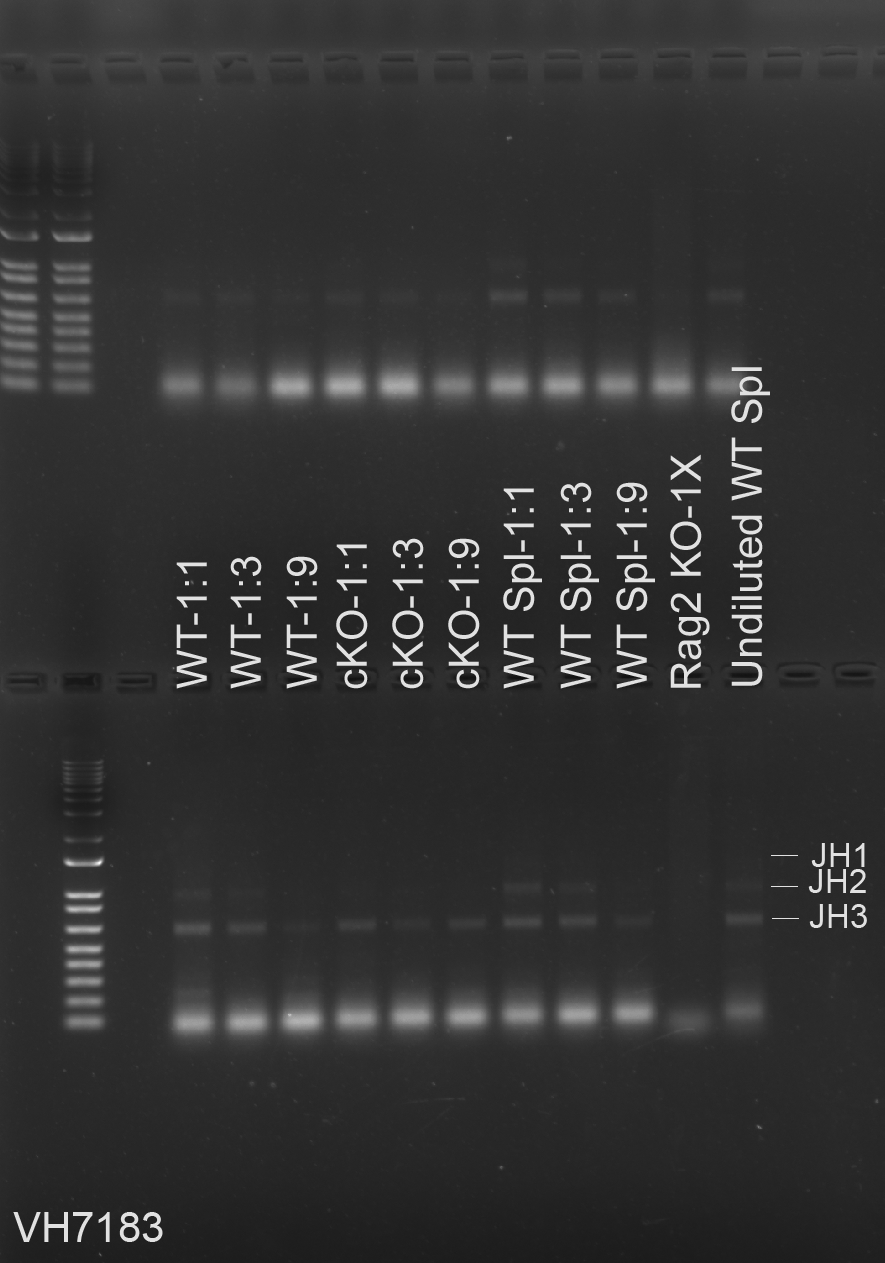

Supplement: Figure 5—source data 1. — This zip archive contains all raw gel images taken for semiquantitative PCR data shown in Figure 5B. Gels were photographed using an Omega Lum G gel imager, which saved the raw image files provided here. Individual files were named based on the VH gene family that was analyzed, and images were saved as full-resolution, 16-bit grayscale TIFF files. In addition to the unedited gel images, a labeled image is provided (named as ‘labeled’) for each gel. [file elife-69621-fig5-data1.zip › Source Data/VH7183 (bottom gel)_labeled.tif]

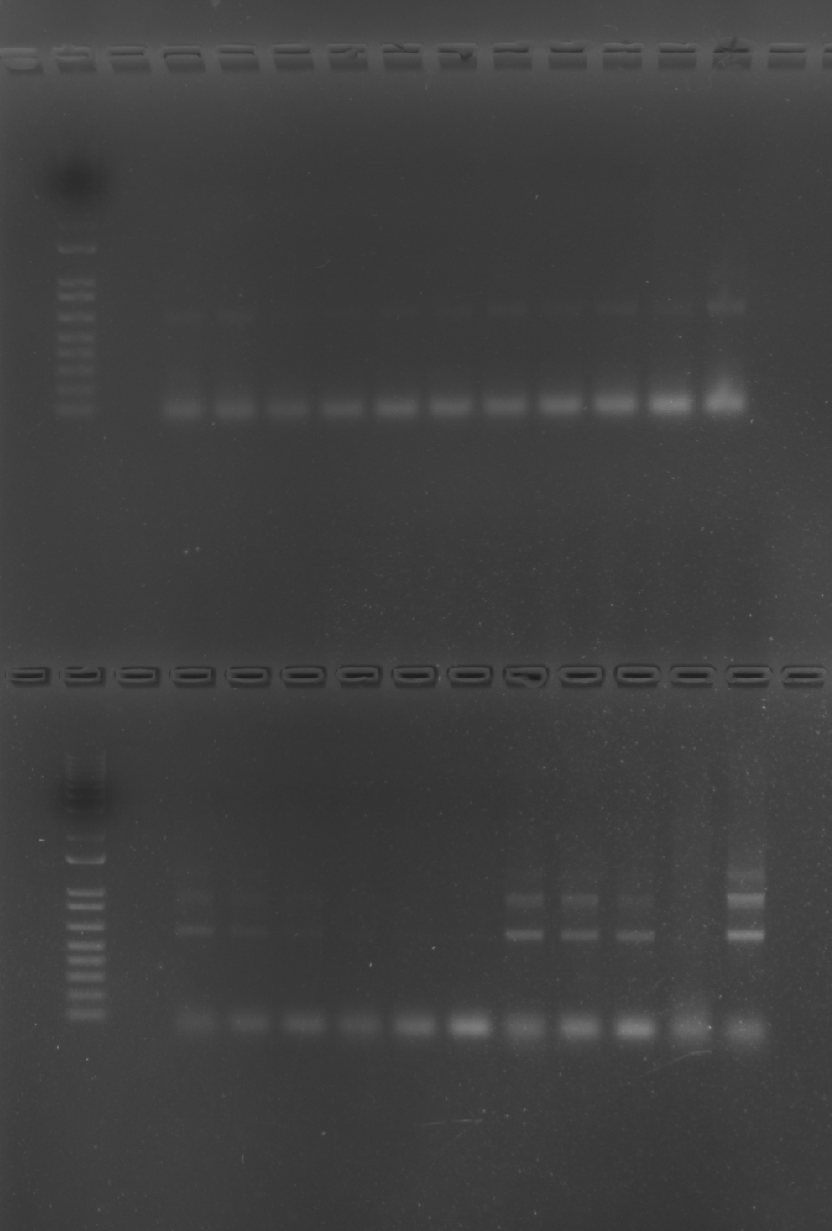

Supplement: Figure 5—source data 1. — This zip archive contains all raw gel images taken for semiquantitative PCR data shown in Figure 5B. Gels were photographed using an Omega Lum G gel imager, which saved the raw image files provided here. Individual files were named based on the VH gene family that was analyzed, and images were saved as full-resolution, 16-bit grayscale TIFF files. In addition to the unedited gel images, a labeled image is provided (named as ‘labeled’) for each gel. [file elife-69621-fig5-data1.zip › Source Data/VHJ558 (bottom gel).tif]

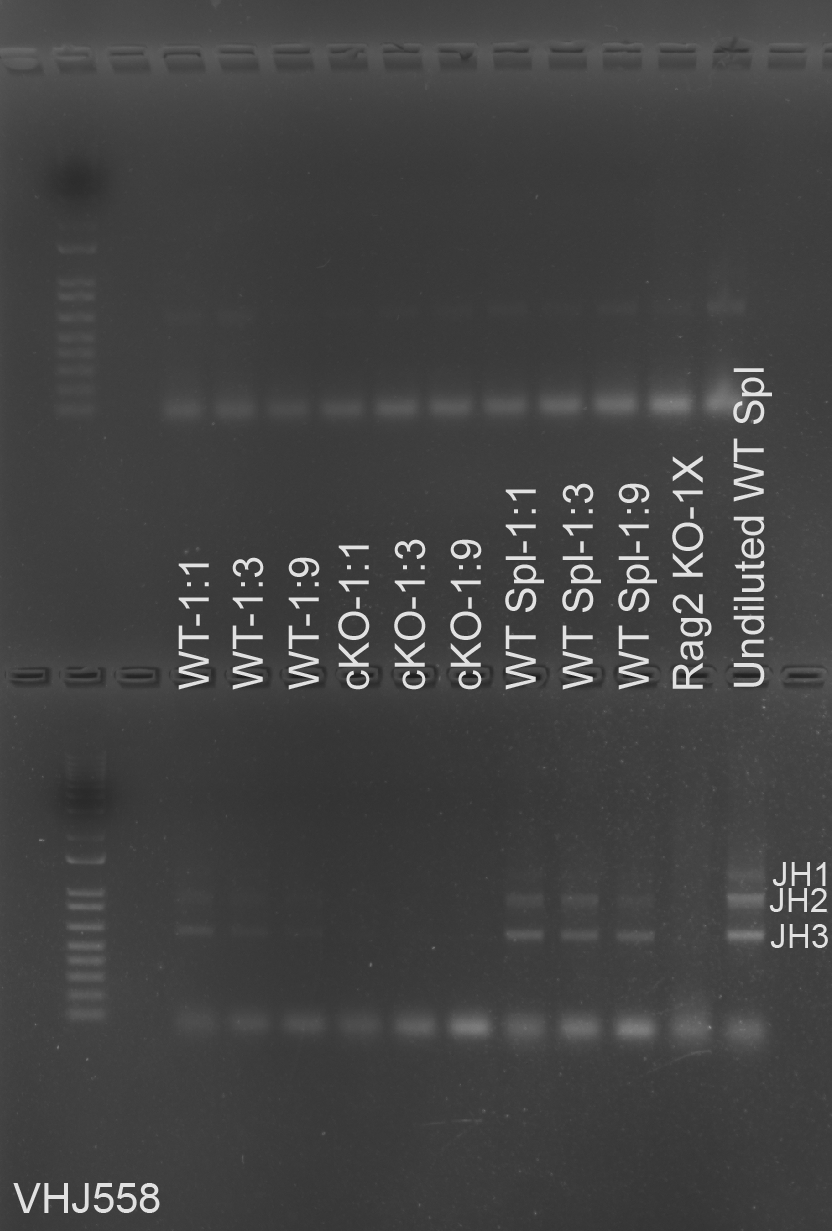

Supplement: Figure 5—source data 1. — This zip archive contains all raw gel images taken for semiquantitative PCR data shown in Figure 5B. Gels were photographed using an Omega Lum G gel imager, which saved the raw image files provided here. Individual files were named based on the VH gene family that was analyzed, and images were saved as full-resolution, 16-bit grayscale TIFF files. In addition to the unedited gel images, a labeled image is provided (named as ‘labeled’) for each gel. [file elife-69621-fig5-data1.zip › Source Data/VHJ558 (bottom gel)_labeled.tif]

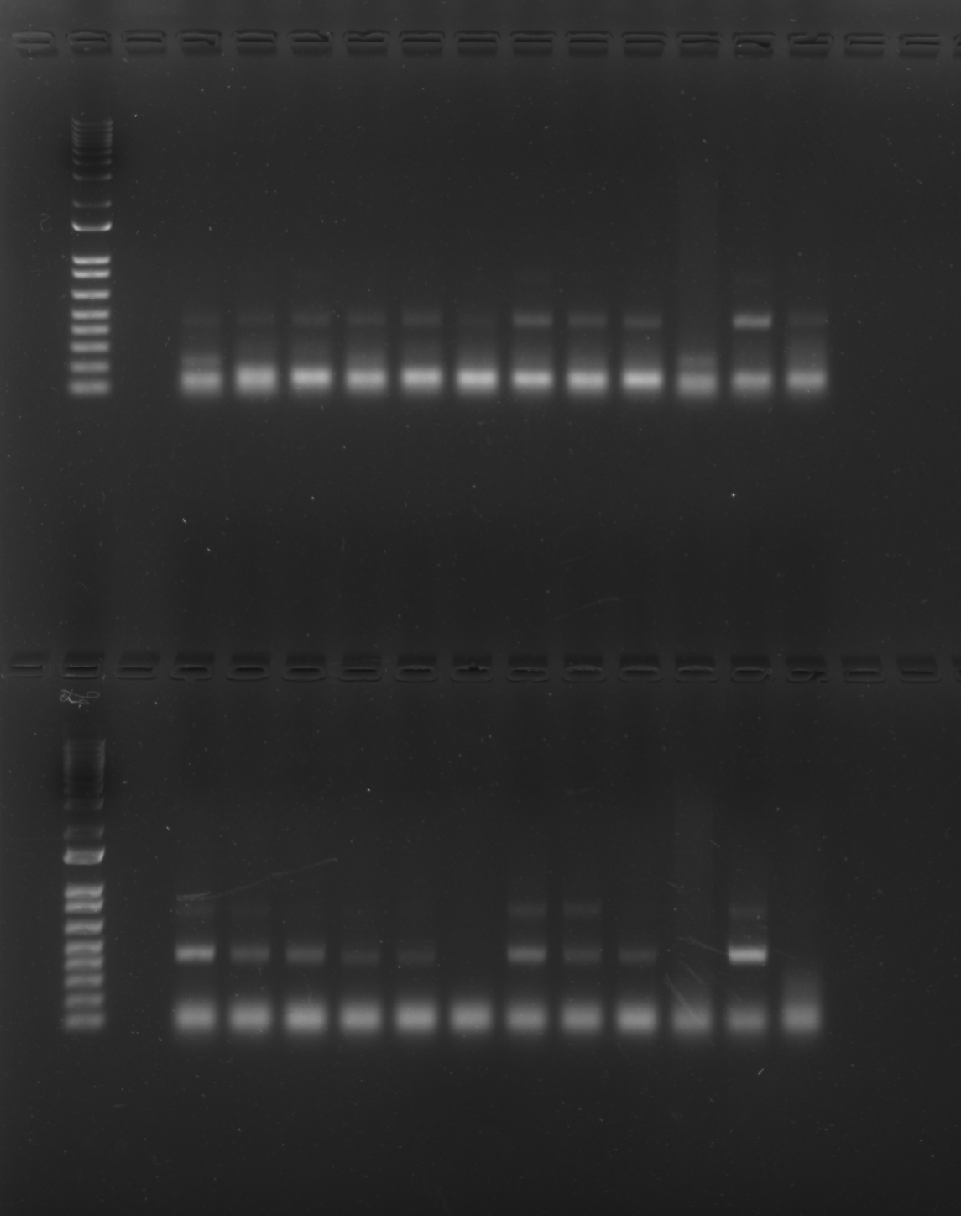

Supplement: Figure 5—source data 1. — This zip archive contains all raw gel images taken for semiquantitative PCR data shown in Figure 5B. Gels were photographed using an Omega Lum G gel imager, which saved the raw image files provided here. Individual files were named based on the VH gene family that was analyzed, and images were saved as full-resolution, 16-bit grayscale TIFF files. In addition to the unedited gel images, a labeled image is provided (named as ‘labeled’) for each gel. [file elife-69621-fig5-data1.zip › Source Data/VHVgam3.8 (bottom gel).tif]

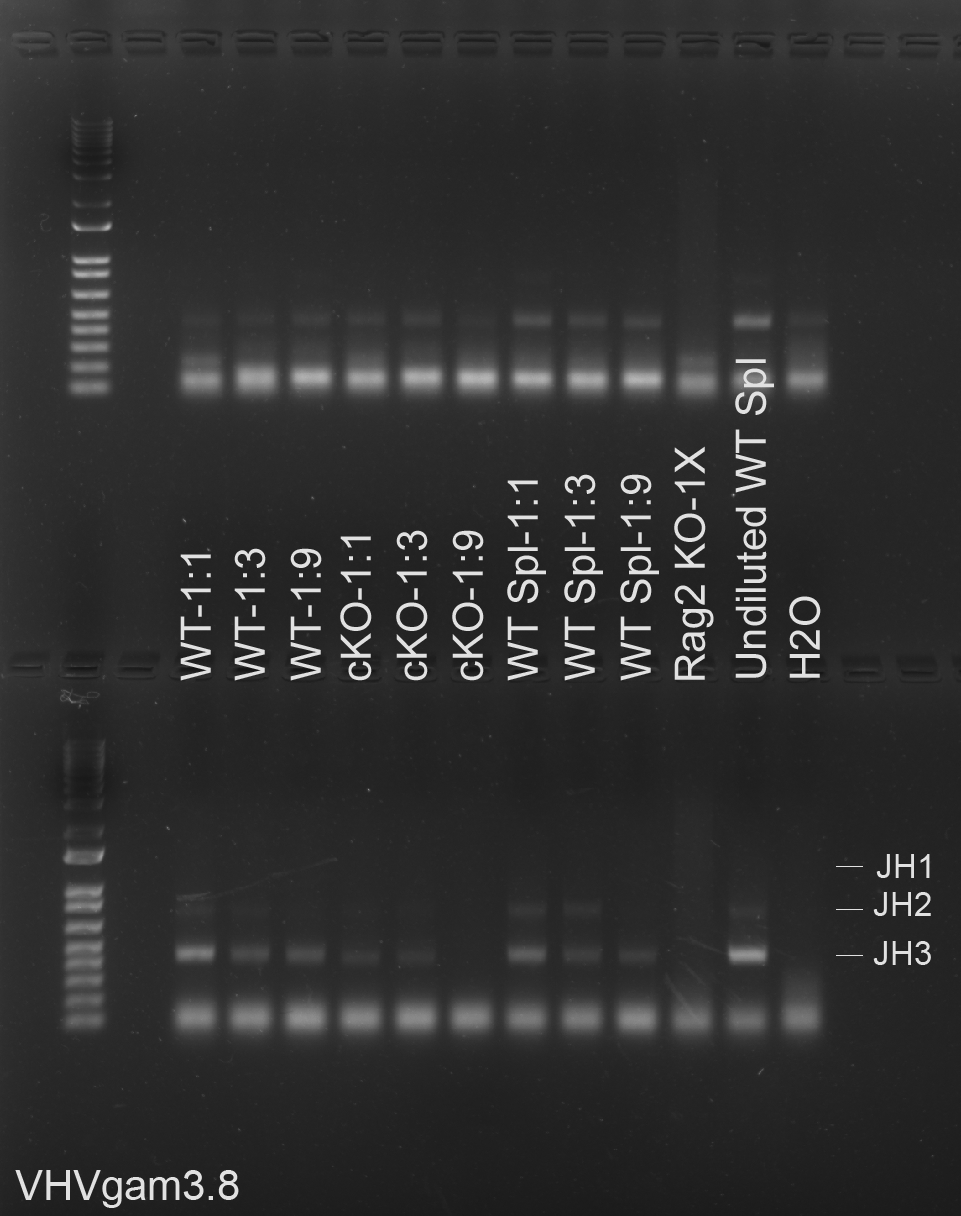

Supplement: Figure 5—source data 1. — This zip archive contains all raw gel images taken for semiquantitative PCR data shown in Figure 5B. Gels were photographed using an Omega Lum G gel imager, which saved the raw image files provided here. Individual files were named based on the VH gene family that was analyzed, and images were saved as full-resolution, 16-bit grayscale TIFF files. In addition to the unedited gel images, a labeled image is provided (named as ‘labeled’) for each gel. [file elife-69621-fig5-data1.zip › Source Data/VHVgam3.8 (bottom gel)_labeled.tif]
